# Assessing Coating With Clove Extract on Improving the Shelf Life of Chicken Fillet at Refrigeration Storage Temperature

**DOI:** 10.1002/fsn3.70786

**Published:** 2025-08-12

**Authors:** Zahra Khoshdouni Farahani, Peyman Mahasti Shotorbani, Afshin Akhondzadeh Basti, Hassan Hamedi, Bahram Hassani, Abdorreza Mohammadi Nafchi

**Affiliations:** ^1^ Department of Food Science and Technology, Faculty of Agriculture Sciences and Food Industry, Science and Research Branch Islamic Azad University Tehran Iran; ^2^ Department of Food Quality Control and Hygiene, Science and Research Branch Islamic Azad University Tehran Iran; ^3^ Department of Food Hygiene, Faculty of Veterinary Medicine University of Tehran Tehran Iran; ^4^ Department of Food Industry, Faculty of Agriculture Ferdowsi University of Mashhad Mashhad Iran; ^5^ Food Technology Division, School of Industrial Technology Universiti Sains Malaysia Penang Malaysia; ^6^ Department of Food Science and Technology, Damghan Branch Islamic Azad University Damghan Iran

**Keywords:** chicken meat, gas chromatography, immersion coating, lipid oxidation, microbial spoilage, plant bioactive compounds, Syzygium aromaticum

## Abstract

Clove extract was used to prevent lipid oxidation and microbial spoilage of chicken fillet in this research. The purpose of this study was to use 0%, 1%, 2%, and 3% concentrations of clove extract to increase the shelf life of chicken fillet at a temperature of 4°C ± 1°C for 7 days. Assessment of chemical spoilage indexes such as pH, peroxide value, thiobarbituric acid, and TVN, as well as microbial parameters (total plate count and Psychrotrophic count) was carried out in 0, 3, and 7 days, and sensory evaluation of chicken fillet (smell, texture, taste, color and total acceptability) was done. The outcomes revealed that 3% clove extract has significantly lower chemical and microbial indicators than the control group (*p* < 0.01). Furthermore, the treatment of 3% clove extract significantly had the lowest pH, peroxide value, thiobarbituric acid, TVN, total plate count, and psychrotrophic count, which were 6.25, 1.56 (meq/kg), 0.39 (mg MDA/kg), 17.65 (mg/100 g), 7.07 (log cfu/g) and 7.29 (log cfu/g), respectively. The control significantly showed the highest pH, peroxide value, thiobarbituric acid, TVN, total plate count, and psychrotrophic count, which were 6.87, 3.43 (mEq/kg), 1.2 (mg MDA/kg), and 33.5 (mg/100 g), respectively. The highest sensory evaluation score in this research was evaluated for the treatment of 3% clove extract. As a consequence, clove extract may be considered an effective coating tool in preventing the deterioration of chicken fillet quality and thus increasing its shelf life.

## Graphical abstract

The chicken filet were dip coated with a various concentration of clove ethanolic extract. The shelf life was assessed with chemical, microbiological and sensory quality tests. The 3% clove extract improved the shelf life by about during 7 days.

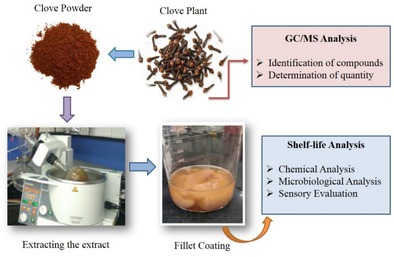

## 1 Introduction

Poultry meat serves as an ideal medium for microbial growth due to its rich nutrient content (Safari et al. 2018). It is widely consumed not only for its distinctive flavor but also as a valuable source of nutrition, providing high‐quality animal proteins, fatty acids, essential minerals, trace elements, and vitamins (Singh et al. 2015). Among all quality attributes, meat color is considered the most critical indicator, as it reflects both freshness and wholesomeness (Shahhoseini et al. 2019). Key factors influencing consumer acceptance and the shelf life of meat include taste, appearance (particularly color), lipid oxidation, and microbial activity (Khoshdouni Farahani, Ebrahimzadeh Mousavi, Seyedain Ardebili, Mohammadi Nafchi, and Paidari 2024). Lipid oxidation is one of the primary causes of meat spoilage, impacting nutritional quality, flavor, odor, and color. The nutritional decline associated with lipid oxidation is mainly attributed to the degradation of essential fatty acids and vitamins, alongside the formation of harmful compounds such as malonaldehyde (Khoshdouni Farahani, Ebrahimzadeh Mousavi, Seyedain Ardebili, Bakhoda, et al. 2024).

Many factors influence lipid oxidation, including light, temperature, oxygen concentration, the presence of antioxidants (Khoshdouni Farahani 2021), enzymes, prooxidants, metal ions such as iron, heme pigments, and mechanical processes (Singh et al. 2015). To counteract these effects, antioxidant and antimicrobial agents are commonly employed (Khoshdouni Farahani and Khoshdouni Farahani 2017). Synthetic chemicals such as tertiary butyl hydroquinone, butylated hydroxyanisole, and butylated hydroxytoluene are widely used for this purpose (Shahosseini et al. 2021). However, concerns regarding the potential harmful effects of these synthetic compounds on human health remain significant (Khoshdouni Farahani and Mousavi 2021).

Recently, research has increasingly focused on identifying natural alternatives with antioxidant and antimicrobial properties. These include cloves, rosemary, ginger, garlic, green tea, cloudberry (Khoshdouni Farahani et al. 2022c), beetroot, willow herb, and cinnamon, among others. Such natural agents can preserve meat quality (Bagheri et al. 2016), prevent economic losses, extend shelf life, and provide consumers with a safer, high‐quality product (Khoshdouni Farahani et al. 2022a). One particularly rich source of natural bioactive compounds is clove, the dried flower bud of *Syzygium aromaticum* . Clove is abundant in polyphenolic compounds and exhibits significant antioxidant and antimicrobial activities, primarily attributed to its main constituent, eugenol (Khoshdouni Farahani et al. 2023a; Singh et al. 2015).

Abdel‐Raouf et al. (2014) investigated the antimicrobial activity of various plant extracts against foodborne bacteria. Baker et al. (2013) evaluated the effects of a combination of rosemary and ginger with sodium lactate on lamb patties stored at −18°C for 150 days, focusing on antioxidant and antimicrobial properties as well as sensory evaluation. Oskoueian et al. (2013) examined the impact of clove essential oil on lipid peroxidation and antioxidant activity in tilapia fish fillets cooked by grilling and microwave methods. Flores et al. (2014) studied the shelf life of raw chicken meat emulsion treated with clove powder, ginger paste, and garlic as natural preservatives under refrigerated conditions (4°C ± 1°C). Similarly, Hosseini et al. (2021) explored the effect of sodium alginate coating containing clove essential oil and lemon extract on extending the shelf life of refrigerated chicken breast. Shukla et al. (2020) also demonstrated that chitosan coatings enriched with clove essential oil significantly improved the quality and shelf life of chicken meat. Given the growing consumer awareness regarding the potential risks of synthetic preservatives, the food industry is increasingly motivated to identify and implement natural alternatives that ensure safety while maintaining product quality (Khoshdouni Farahani et al. 2023b).

Nowadays, considerable research efforts are focused on reducing or eliminating chemical additives in food by replacing them with natural substances. In this context, many studies aim to identify natural antioxidants derived from plants to extend the shelf life and preserve the quality of meat and meat products. These products serve as primary sources of protein, vitamins, and minerals (Khoshdouni Farahani et al. 2021). To enhance storage stability and maintain quality, antimicrobial and antioxidant agents—mostly synthetic—are commonly applied. In line with previous research (Khoshdouni Farahani et al. 2022b), this study investigates the antioxidant and antimicrobial effects of clove ethanol extract on chicken meat stored under refrigerated conditions. To the best of our knowledge, no prior studies have reported on the use of clove ethanol extract for extending the shelf life of poultry meat, particularly chicken fillet, alongside the identification of its volatile compounds extracted by hexane (Farahani 2021b). Therefore, this research aims to evaluate the impact of clove ethanol extract on various chemical, sensory, and microbial properties of chicken fillet during refrigeration, as well as to analyze and identify the volatile compounds in the hexane extract using GC/MS.

## 2 Materials and Methods

### 2.1 Materials

Thiobarbituric acid, catalysts, and microbial culture medium were procured from Merck Co. Ethanol, hexane, and chemical materials were from Merck Germany (Darmstadt). All reagents were analytical grade. Clove and chicken fillets were provided by the local market (Tehran, Iran).

### 2.2 Clove Extract

Fifty grams of dried clove powder were macerated in 500 mL of 70% ethanol (v/v) under continuous mechanical agitation using a rotary shaker at room temperature, protected from light, for 48 h (Farahani 2021c). Following maceration, the mixture was filtered, and the solvent was removed using rotary evaporation at 60°C. The resulting extract was further dried in a vacuum oven at 30°C for two hours to ensure complete removal of residual solvent. The final extract was stored in a dark bottle at 4°C until use (Kim et al. 2013).

### 2.3 Analysis of Compounds by GC/MS

The volatile compounds of the extract were isolated using hexane as the solvent for injection into the GC/MS system. Gas chromatography (GC) analysis was performed on a Hewlett‐Packard GC‐HP‐6890 gas chromatograph equipped with a flame ionization detector and a methyl silicone cross‐linked column (HP‐1MS) measuring 60 m × 0.20 mm i.d. with a 0.25 μm film thickness. The oven temperature was programmed from an initial temperature of 160°C to a final temperature of 230°C at a rate of 7°C/min. The injector temperature was set at 250°C. Helium was used as the carrier gas with a linear velocity of 1 mL/min. Samples were injected in split mode with a split ratio of 1:20. GC/MS analysis was conducted using a Hewlett‐Packard HP‐5970 mass spectrometer. The ion source temperature was maintained at 250°C, with an ionization energy of 70 eV and a scan time of 1 s. Compound identification was carried out by comparing mass spectra and retention times with those of authentic standards whenever available. Additionally, the National Institute of Standards and Technology (NIST) Mass Spectra Library was employed as a reference for compound identification.

### 2.4 Treatment Preparation: Chicken Fillet

Chicken fillet samples (50 g) were immersed in ethanolic clove extract at concentrations of 0%, 1%, 2%, and 3% for 30 min. The surface area to volume ratio of solution to sample is also about 1:5 (cm^2^/mL) so that there is complete immersion. After immersion, the samples were dried in ambient air for 5–10 min and then placed in a refrigerator (4°C) to stabilize the coating. Chicken fillet samples are packed tightly in plastic bags and kept at refrigerator temperature (4°C ± 1°C) for 10 days. Treatments are shown in Table 1.

**TABLE 1:** Chicken fillet treatments coated with clove extract.

Treatments	Clove extract (%)	Temperature (°C)
1	0	4 ± 1
2	1	4 ± 1
3	2	4 ± 1
4	3	4 ± 1

### 2.5 Chemical Analysis

The protein, fat, and moisture content of the chicken fillet was determined according to Iranian National Standards.

### 2.6 Moisture Content

The empty crucible was dried at 100°C for 1 h and cooled in a desiccator (Farahani 2021d). Then, 5–8 g of a uniform sample was weighed into the crucible (A), dried at 100°C for 4 h, weighed (B), dried for one more hour, and weighed again. Moisture content was calculated using Equation 1 (Khoshdouni Farahani et al. 2022c):

(1) $$\text{Moisure Content}=\frac{\left(A-B\right)\times 100\;}{\text{Weight}\;}$$

### 2.7 Protein Content

Protein content was determined using the macro‐Kjeldahl method, based on total nitrogen measurement. Approximately 2 g of the homogeneous sample was weighed into a digestion flask. Then, 20 mL of sulfuric acid and 8 g of catalyst mixture (96% potassium sulfate, 3.5% copper sulfate, 0.5% selenium oxide) were added. The flask was connected to the digestion apparatus and gradually heated until a nearly colorless solution was obtained. After digestion, the contents were diluted and transferred to the distillation apparatus.

During distillation, 50 mL of boric acid solution with a few drops of methyl red indicator was placed in the receiving flask. A 50% sodium hydroxide solution was added to create an alkaline environment, allowing ammonia to be distilled and collected in the boric acid solution. Approximately 200–250 mL of distillate was collected. The solution was then titrated with 1 N sulfuric acid. A blank control was performed simultaneously without the sample. Considering that 1 mL of sulfuric acid corresponds to 0.0014 g of nitrogen, the protein percentage was calculated according to Equation 2 (Farahani et al. 2013):

(2) $$\text{Protein Percent}=\frac{\left(V1-V2\right)\times 0/0014\times 6/25\times 100\;}{m}$$

where, V1, The amount of milliliters of sulfuric acid used for the sample; V2, The amount of milliliters of sulfuric acid used for the control; W, Sample amount (grams).

### 2.8 Fat Content

Three to five grams of the homogenized sample were placed at 100°C for 6 h to completely evaporate moisture. The extraction flask was dried in an oven, cooled in a desiccator, and weighed. The sample was then transferred into a thimble and placed in the Soxhlet extraction apparatus. The apparatus was accurately weighed (A), and 250 mL of chloroform was added. The system was connected to the distillation setup; the cooling water valve was opened, and extraction continued until all fat was extracted. Subsequently, the chloroform was completely evaporated from the flask using a rotary evaporator or oven, and the flask was weighed again (B). The total fat content was calculated using the following equation (Equation 3) (Farahani et al. 2013):

(3) $$\mathrm{Fat}\;\text{Percent}=\frac{\left(B-A\right)\times 100\;}{m}$$

where, A, weight of the empty balloon (grams); B, balloon weight plus fat (grams); m, Sample weight (grams).

### 2.9 pH Measurement

An electric PH meter is used to measure pH. First, some of the sample was homogenized; then 10 g of the sample was mixed with 90 mL of distilled water and calibrated with buffer solutions 4 and 7 to use this device (Farahani et al. 2013). After calibrating, the electrode of the device was distilled twice with distilled water, washed, and dried with a paper towel; then the electrode was placed in the sample and its pH was read (Khoshdouni Farahani et al. 2023b).

### 2.10 Fat Extraction and Peroxide Index Determination

#### 2.10.1 Fat Extraction

50 g of chicken fillet was dissolved in 150 mL of chloroform and methanol solution (50 mL of chloroform and 100 mL of methanol) in a blender. Then 50 mL of chloroform and 50 mL of distilled water were added to it, and the mixture was homogenized for 1 min. After that, the resulting mixture was filtered for 5 min in a Buchner funnel under vacuum. Then the mixture was poured into the decanter until it became two phases. After that, the oily phase (lower phase) was collected, and the sample solvent was evaporated by rotary (at 60°C and 337 vacuum) and finally, the obtained oil was used for peroxide determination test (Khoddami et al. 2012).

#### 2.10.2 Peroxide Value

The peroxide value was determined using the IDF standard method 74A:1991. To prepare the ferrous chloride solution, 0.4 g of barium chloride dihydrate was dissolved in 50 mL of distilled water. This solution was slowly added, under constant stirring, to a ferrous sulfate solution prepared by dissolving 0.5 g of FeSO_4_·7H_2_O in 50 mL of water. Subsequently, 2 mL of 10 N hydrochloric acid was added. The resulting barium sulfate precipitate was filtered out, yielding a clear ferrous ion solution, which was stored in a brown glass bottle, protected from light.

For the preparation of the ammonium thiocyanate solution, 30 g of ammonium thiocyanate was dissolved in distilled water and diluted to a final volume of 100 mL. To determine the peroxide value, the sample was placed in a glass tube and mixed with 9.8 mL of a chloroform–methanol solution (3:7 v/v) using a vortex mixer for 2–4 s. Then, 50 μL of the ammonium thiocyanate solution was added and vortexed again for 2–4 s. Next, 50 μL of the ferrous ion solution was added, followed by another vortexing step for 2–4 s. After incubation at room temperature for 5 min, the absorbance was measured at 500 nm using a spectrophotometer, with a blank sample containing all reagents except the test sample used as the control. The entire procedure was completed within 10 min. The peroxide value was expressed as milliequivalents of peroxide per kilogram of sample and calculated using the following formula (Achlina and Nurazizah 2024):

(4) $$\text{Peroxide index}=\frac{\left(\mathrm{As}-\mathrm{Ab}\right)\times m}{55.84\times m\times 2}$$

where, A_S_, Sample absorption; A_b_, Absorbance blank; m, the slope of the calibration graph, which here is equal to 41.52; m_0_, Sample weight (grams); 2, To express the peroxide index as milliequivalents of peroxide instead of milliequivalents of oxygen.

#### 2.10.3 TVN

Volatile nitrogen compounds in meat samples, which are formed through the decomposition of protein molecules, were measured using the macro‐Kjeldahl method. A total of 10 g of the homogenized sample was placed into a Kjeldahl digestion flask along with 2 g of magnesium oxide (as a catalyst), 300 mL of distilled water, and a few glass beads. The flask was connected to the rest of the Kjeldahl apparatus, and cold water circulation was initiated. In the receiving flask (Erlenmeyer), 25 mL of 2% boric acid solution and a few drops of 2% methyl red indicator were added. The tip of the condenser was immersed in this solution, causing the solution to initially appear red. The digestion process was carried out by boiling the sample for 10 min, followed by a distillation step that lasted 25 min. During this process, volatile nitrogen compounds released from the meat were distilled and absorbed in the boric acid solution, turning the solution yellow due to the alkaline nature of the distillate. After distillation, the receiving solution was titrated with 0.1 N sulfuric acid until a red endpoint was observed. Each milliliter of 0.1 N sulfuric acid corresponds to 0.0014 g (or 1.4 mg) of nitrogen. The amount of volatile nitrogen was then calculated and expressed in milligrams per 100 g of sample using the following formula (Equation 5) (Delavari et al. 2015; Gheibi et al. 2023).

(5) $$\left(\frac{\mathrm{mg}}{100\mathrm{gr}}\right)\mathrm{TVN}=\frac{\;V\times 14\times N\times 100\;}{\;m}$$

where, N, Normality of sulfuric acid consumption; V, Sulfuric acid consumption volume; m, Sample weight (grams).

### 2.11 Evaluation of Thiobarbituric Acid

Lipid oxidation in chicken fillet was monitored by measuring thiobarbituric acid reactive substances (TBARS) at 0, 3, and 7 days during refrigerated storage. The production of TBA was measured spectrophotometrically at 532 nm and expressed as milligrams of malondialdehyde (MDA) per kilogram of sample. For the TBA assay, 200 mg of the sample was weighed and placed into a 25 mL volumetric flask. The sample was dissolved in a small volume of 1‐butanol and then brought to volume with the same solvent. If the solution was not completely clear, it was filtered. Five milliliters of the resulting sample solution were transferred to a capped test tube, to which 5 mL of the TBA reagent was added. The TBA reagent was prepared by dissolving 200 mg of thiobarbituric acid in 100 mL of 1‐butanol and filtering the solution before use. The sealed test tubes were incubated in a water bath at 95°C for 120 min. After incubation, the tubes were cooled under running water for 10 min until they reached room temperature. The absorbance of the reaction mixture was measured at 530 nm using distilled water as a blank. The TBA value was calculated using the following formula (Equation 6) (Kelany et al. 2024):

(6) $$\mathrm{TBA}=\frac{50\times \left(A-B\right)}{m}$$

where, A, The absorption rate of the test solution; B, absorption rate of reactive control; m, Test mass, in grams.

The number 50 is a valid coefficient in the condition that the volume of the volumetric balloon is 25 mL, and the tube width is 10 mm.

### 2.12 Microbial Analysis

#### 2.12.1 Total Plate Count

The total count was determined by pouring 10 g of sample into 90 mL of 0.85% NaCl solution. The sample was homogenized, and from this dilution, other decimal dilutions were prepared and cultured in the appropriate culture. Meanwhile, the total Plate Count (TPC) was determined using plate count agar (PCA, Merck) after incubation for 72 h at 32°C (Tiesler and Miller 2023) and the microbiological data were converted into logarithms of the number of colony units (CFU/g).

#### 2.12.2 Psychrotrophic Bacteria Count

**FIGURE 1:**
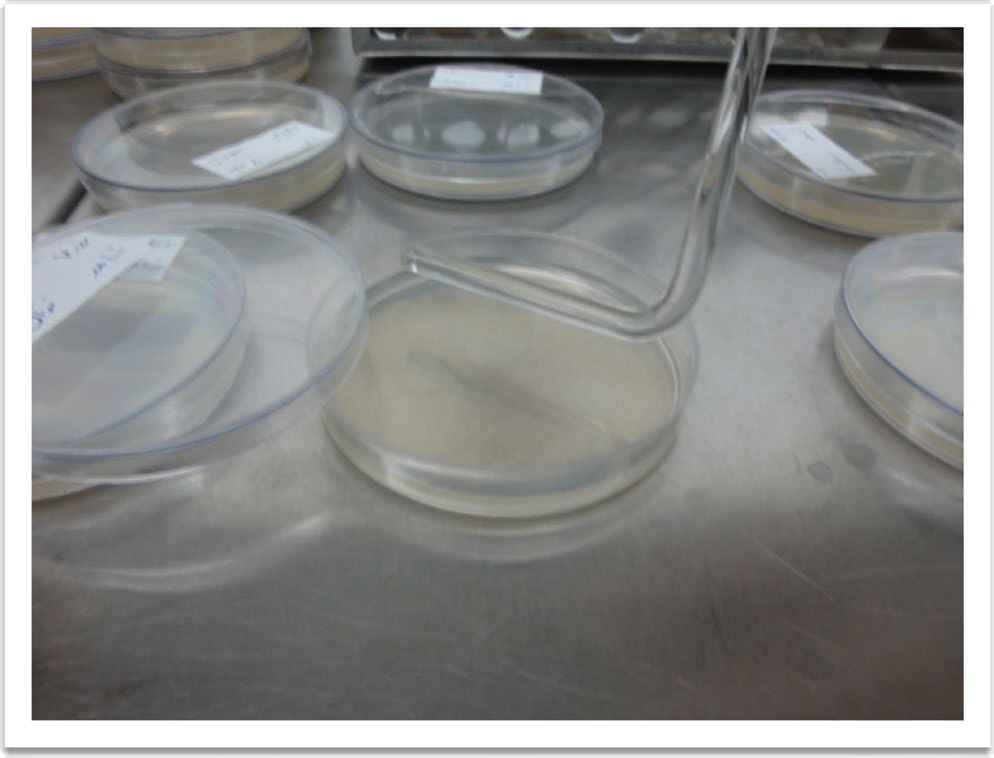
Surface culture method.

To check the number of Psychrotrophic bacteria, 10 g of the samples were poured into 90 mL of 0.1% peptone water (diluting solution) and homogenized by stomacher. From this initial dilution, other decimal dilutions were prepared to be cultured in the culture medium. The number of Psychrotrophic bacteria was determined using Plate Count Agar (PCA, Merck) after incubation for 10 days at 7°C (Cha et al. 2023). The resulting data was transformed into the logarithm of the number of colony‐forming units (CFU/g) (Figure 1).

#### 2.12.3 Sensory Evaluation

Sensory evaluation was done only by the 5‐point hedonic method, and Table 2 shows an example of it. The characteristics of cooked samples (i.e., smell, taste, color, texture and overall acceptance) were evaluated using a trained panel of 10 members. The treated chicken fillet was microwaved and immediately served to the panelists. The participant scored the attributes, where 1 = unacceptable, 5 = very acceptable, while the limit of acceptance was 3. Sensory evaluation was done in 1 day of sampling (Mostafa et al. 2023). It is noted that participants in the sensory study gave their consent to take part and that the appropriate protocols for protecting the rights and privacy of all participants were utilized during the execution of the research. Moreover, ethical consent was obtained verbally and in writing from the subjects.

**TABLE 2:** Sensory evaluation by 5‐point hedonic.

			Score		
Sensory Index	1	2	3	4	5
Texture					
Color					
Flavor					
Taste					
Total acceptance					

### 2.13 Statistical Analysis

For microbiological and chemical analyses, samples were prepared and analyzed in triplicate (*n* = 3) for each treatment group at each storage time point. Each replicate represented an independent portion of the sample to ensure accuracy and reproducibility of results; mean values ± standard deviations were reported for each case. The one‐sample Kolmogorov–Smirnov test was employed to determine the normal distribution of data. Analysis of data for all treatments of clove extract was done by one‐way ANOVA (SPSS 22). The Duncan test was used to determine significant differences between treatments. The Friedman test was performed to evaluate the significance of differences between sensory values. A probability level of *p* < 0.01 was used in testing the statistical significance of all experimental data.

## 3 Results and Discussion

### 3.1 Analysis of Compounds of Extract by GC/MS

**FIGURE 2:**
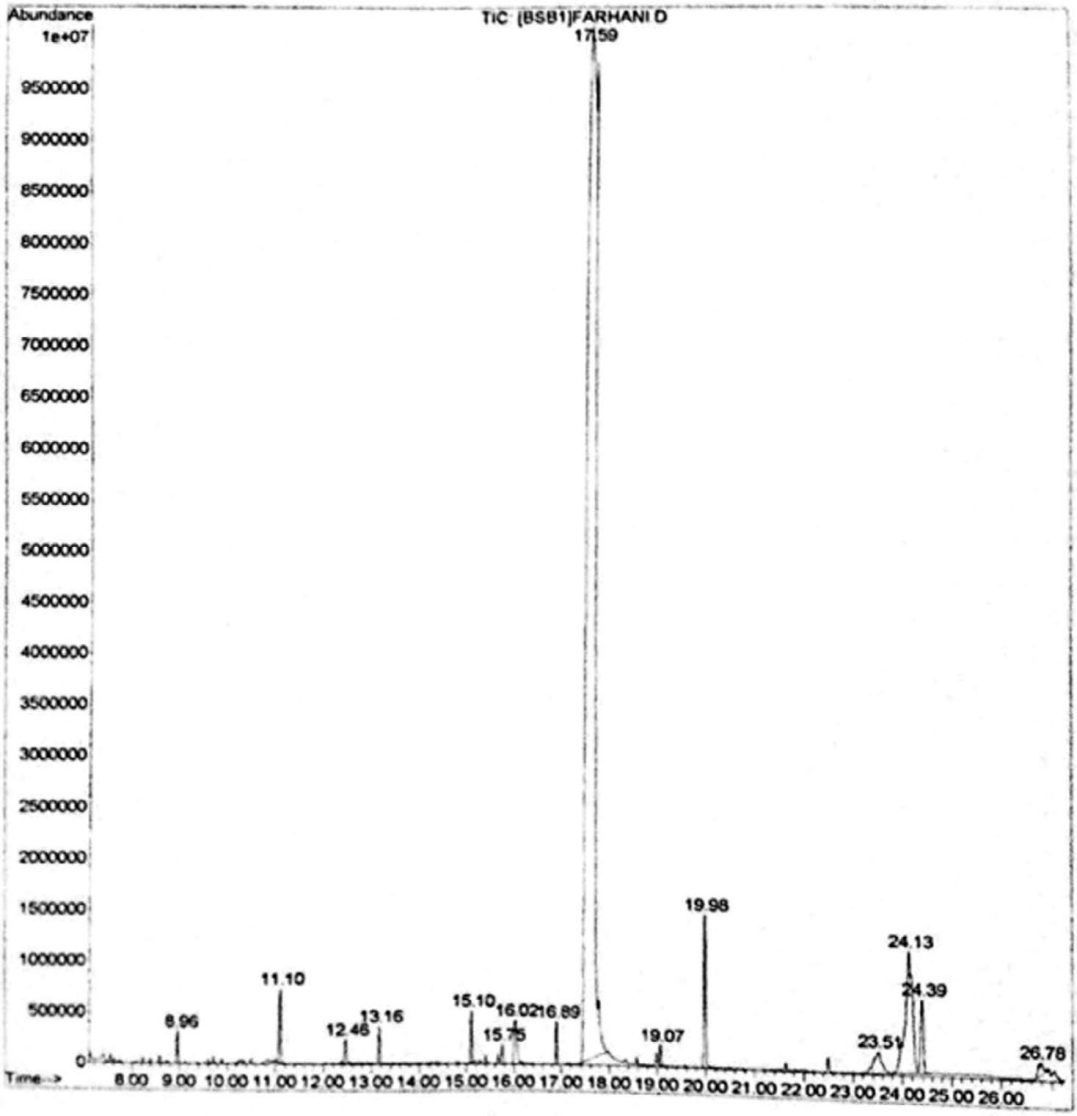
Chromatogram of clove extract.

Analysis of the data reveals that the predominant compound in the clove extract is eugenol, constituting approximately 84.5%, followed by eugenol acetate (2.24%), toluene (1.89%), heptacosane (1.6%), and chavicol (1.22%). It is important to note that the chemical profile of clove extracts can vary significantly depending on geographic origin and the extraction solvents employed, as demonstrated in Table 3 and Figure 2 (El Ghallab et al. 2023; Fathi et al. 2012).

**TABLE 3:** Constituents of the extract, content, and retention time.

Peak no.	Name of the compound	Conc. %	Retention time (min)
1	Alfa‐pinene	0.02	3.98
2	Toluene	1.89	4.68
3	cymene	0.02	4.92
4	2‐Pentanone‐3‐methylene	0.15	5.23
5	2‐heptyle acetate	0.05	5.67
6	2‐Pentanone, 4‐hydroxy‐4‐methyl	0.85	5.88
7	m‐Dioxan‐4‐ol, 2,6‐diethyl‐5methyl acetate	0.09	6.73
8	Beta‐Ocimene	0.5	7.12
9	2,4,4,6‐Tetramethyl‐4,5‐dihydro‐1,3‐oxazine	0.15	7.84
10	2‐Nonane	0.05	8.35
11	Linalool	0.03	8.46
12	Decane	1.02	11.10
13	Methyl Salicylate	0.09	11.23
14	Phenol,2‐methoxy	0.45	12.46
15	2,4,4,6‐Tetramethyl‐4,5‐dihydro‐1,3‐oxazine	0.12	13.33
16	Naphthalene,decahydro‐1,5‐dimethyl	0.42	15.75
17	Chavicol	1.22	16.03
18	Copaene	0.23	17.87
19	Eugenol	84.50	17.59
20	Trans‐Beta‐Caryophyllene	0.45	19.07
21	Eugenyl Acetate	2.24	19.98
22	1,4,7‐Cycloundecatriene, 1,5,9,9‐tetramethyl‐,Z,Z,Z—	0.32	20.01
23	alpha‐Farnesene	0.13	20.64
24	Alfa‐Humulene	0.34	21.24
25	Cadinene	0.05	22.15
26	2′, 3′,4′ Trimethoxyacetophenone	0.32	22.49
27	Heptacosane	1.62	23.51

The variability in essential oil and extract composition across different ecological regions is well‐documented. This variation arises not only from environmental factors but also from intrinsic plant physiology, which can fluctuate based on harvest time and developmental stage. Optimal harvest timing is critical, as it directly affects the concentration and efficacy of bioactive constituents.

Moreover, both external environmental parameters such as temperature, humidity, light exposure, geographic location, and soil composition and internal plant factors collectively influence the yield and chemical profile of essential oils and plant extracts. Therefore, selecting an appropriate extraction method and solvent is paramount for maximizing the recovery of desired phenolic compounds. This consideration ensures that the bioactivity and potency of the extracts align with the intended application (El Ghallab et al. 2023; Farahani 2021a). In summary, the interplay between ecological, physiological, and methodological factors must be thoroughly understood and controlled to obtain clove extracts with consistent and potent bioactive profiles.

### 3.2 Chemical Analysis

The moisture, fat, and protein content of the chicken fillet were determined to be 2%, 18.75%, and 73.2%, respectively. Chicken meat is widely recognized as an important source of high‐quality protein. However, reported values for fat, protein, and moisture content vary considerably across studies. These variations can be attributed to several factors, including the genetic breed of the poultry, rearing conditions, geographical location, diet composition, and environmental influences. Additionally, the proportion of dietary protein and fat plays a crucial role in determining the nutritional profile of chicken meat (Hematizad et al. 2021). Understanding these variables is essential for interpreting compositional differences and for optimizing poultry production to meet specific nutritional standards.

### 3.3 pH

The changes in pH values of the treatments during storage are summarized in Table 4. The pH of chicken fillet during storage is primarily influenced by the breakdown of nitrogenous compounds and microbial activity, particularly proteolytic bacteria (Michalcyzk et al. 2012). As nitrogenous compounds decompose over time, pH typically increases due to the accumulation of basic metabolites such as ammonia. This rise in pH is often associated with the growth of proteolytic microorganisms. In the present study, pH values generally increased over the storage period; however, treatments containing clove extract, especially at higher concentrations, exhibited a significantly lower pH compared to the control. This suggests that the extract may inhibit proteolytic bacterial activity, thereby reducing the pH elevation.

**TABLE 4:** The measurement pH values of chicken fillets during a 7‐day storage period.

Treatments	Storage time (day)		
	0	3	7
1	5.9700 ± 0.042 (ab)	6.2750 ± 0.035 (c)	6.8750 ± 0.035 (f)
2	5.9700 ± 0.042 (ab)	6.050 ± 0.049 (b)	6.7150 ± 0.049 (e)
3	5.9700 ± 0.042 (ab)	5.9700 ± 0.042 (ab)	6.5100 ± 0.056 (d)
4	5.9700 ± 0.042 (ab)	5.8600 ± 0.056 (a)	6.2550 ± 0.063 (c)

*Note:* Values are mean ± SD from triplicate determinations; Different letters show the statistical significant differences (*p* < 0.01).

Our observations align with those reported by Chen et al. (2023), who investigated the effect of oregano extract on minced beef stored for 10 days at 4°C. They observed an initial pH of 5.97 ± 0.04, which increased to 6.88 ± 0.04 by the end of storage, demonstrating a similar trend of pH increase during storage. Arumugham et al. (2023) studied the antimicrobial effects of various spices on chicken over 15 days, noting a comparable pH increase attributed to proteolytic bacterial metabolism of amino acids. These findings collectively highlight the role of plant extracts in moderating microbial activity and pH changes during meat storage.

### 3.4 Peroxide Value

The results of the peroxide value (PV) analysis of lipids during refrigerated storage are presented in Table 5. The peroxide value is a widely used indicator of the primary stage of lipid oxidation, representing the concentration of hydroperoxides formed during early oxidative degradation. Although peroxides are tasteless and odorless and not directly detectable by consumers, their accumulation is a precursor to the development of off‐flavors and rancidity (Ozyurt et al. 2012).

**TABLE 5:** The measurement peroxid values (meq/kg) of chicken fillets during a 7‐day storage period.

Treatment	Storage time (day)		
	0	3	7
1	1.2550 ± 0.077 (b)	2.7550 ± 0.077 (e)	3.4300 ± 0.056 (f)
2	1.2550 ± 0.077 (b)	1.5750 ± 0.106 (c)	2.7600 ± 0.084 (e)
3	1.2550 ± 0.077 (b)	1.5550 ± 0.077 (c)	2.4600 ± 0.084 (d)
4	1.2550 ± 0.077 (b)	0.9450 ± 0.056 (a)	1.5600 ± 0.084 (c)

*Note:* Values are mean ± SD from triplicate determinations; Different letters show the statistical significant differences (*p* < 0.01).

In this study, peroxide values gradually increased in all treatments over the course of storage, consistent with oxidative progression. This trend is in agreement with the findings of Safari et al. (2018), who also reported increasing PVs in refrigerated meat products. However, significant differences (*p* < 0.01) were observed between treatments. Notably, the sample treated with 3% clove extract consistently exhibited the lowest peroxide values throughout the storage period. After 72 h, this treatment even showed a lower peroxide value than the initial PV of the control sample, suggesting strong antioxidant activity from the clove extract. The initial PV across samples was approximately 1.26 ± 0.08 meq/kg. By Day 7, the control group recorded the highest peroxide value, reflecting extensive lipid oxidation, while the 3% clove extract treatment maintained the lowest PV at 1.56 ± 0.08 meq/kg. These results align with previous studies such as Ozyurt et al. (2012) and Gómez‐Estaca et al. (2017), which confirmed the antioxidant capacity of clove extract in retarding lipid peroxidation.

The antioxidant effect of clove extract is attributed to its high content of phenolic compounds, particularly eugenol, which acts as a free radical scavenger. Increasing extract concentration was correlated with greater inhibition of peroxide formation, supporting the use of clove extract as a natural antioxidant to enhance the shelf life and oxidative stability of meat products. At the end of the storage time, the best results were related to 3% clove extract with a peroxide value of 1.5600 ± 0.084 meq/kg (Safari et al. 2018).

### 3.5 TBA

The results of oxidative lipid degradation during refrigerated storage, assessed by thiobarbituric acid (TBA) values, are presented in Table 6. TBA is a well‐established indicator for evaluating secondary lipid oxidation by measuring malondialdehyde (MDA), a major aldehyde produced during the breakdown of polyunsaturated fatty acids (Wang et al. 2016). In this study, TBA values increased progressively in all treatment groups throughout the storage period, reflecting ongoing lipid oxidation, consistent with findings reported by Safari et al. (2018).

**TABLE 6:** The measurement TBA values (mg MDA/kg) of chicken fillets during a 7‐day storage period.

Treatment	Storage time (day)		
	0	3	7
1	0.4340 ± 0.018 (bc)	0.8080 ± 0.022 (f)	1.2710 ± 0.026 (g)
2	0.4340 ± 0.018 (bc)	1.4600 ± 0.028 (c)	0.6615 ± 0.019 (e)
3	0.4340 ± 0.018 (bc)	0.3710 ± 0.026 (b)	0.5295 ± 0.019 (d)
4	0.4340 ± 0.018 (bc)	0.2080 ± 0.016 (a)	0.3985 ± 0.019 (bc)

*Note:* Values are mean ± SD from triplicate determinations; Different letters show the statistical significant differences (*p* < 0.01).

Statistical analysis revealed that TBA values were significantly influenced by the presence of 3% clove extract during refrigeration (*p* < 0.01). Initially, all samples exhibited low TBA values, averaging 0.4340 mg MDA/kg after 72 h; however, the treatment containing 3% clove extract consistently demonstrated lower TBA values compared to other treatments, indicating a protective antioxidant effect. These findings are in agreement with previous research by Iheagwara (2013) and Safari et al. (2018), which confirmed the effectiveness of clove extract in mitigating lipid peroxidation.

The antioxidant properties of clove extract are primarily attributed to its phenolic constituents, such as eugenol, which act as free radical scavengers by interrupting the propagation of free radical chains. This antioxidant mechanism underpins its widespread application in food preservation, particularly in delaying oxidation in vegetable oils and animal fats (Iheagwara 2013). At the conclusion of the 7‐day refrigeration period, the control samples exhibited the highest TBA values, indicative of greater lipid degradation (*p* < 0.01) (Safari et al. 2018). Importantly, the degree of lipid oxidation inhibition improved with increasing concentrations of clove extract. The best protective effect was observed in the 3% clove extract treatment, which recorded a TBA value of 0.3985 mg MDA/kg in chicken fillet at the end of storage, reinforcing the extract's efficacy as a natural antioxidant (Iheagwara 2013; Safari et al. 2018).

### 3.6 TVN

The results of the Total Volatile Nitrogen (TVN) analysis from refrigerated treatments are presented in Table 7. The TVN index, which comprises compounds such as trimethylamine, dimethylamine, ammonia, and other volatile nitrogenous substances, serves as a reliable indicator of meat spoilage and is closely linked to the metabolic activity of spoilage bacteria (Raeisi et al. 2019).

**TABLE 7:** The measurement TVN values (mg/100 g of the sample) of chicken fillets during a 7‐day storage period.

Treatment	Storage time (day)		
	0	3	7
1	14 ± 1.13 (a)	23.0500 ± 1.060 (de)	33.5000 ± 1.838 (g)
2	14 ± 1.131 (a)	20.6000 ± 0.565 (cd)	28.6500 ± 0.777 (f)
3	14 ± 1.131 (a)	19.2500 ± 0.494 (c)	24.2500 ± 0.777 (e)
4	14 ± 1.131 (a)	15.1000 ± 0.424 (ab)	17.6500 ± 0.777 (bc)

*Note:* Values are mean ± SD from triplicate determinations; Different letters show the statistical significant differences (*p* < 0.01).

On day zero, the initial TVN value was measured at 14 mg per 100 g of sample. According to standards set by the Iranian Veterinary Organization, the permissible limit for volatile nitrogen in chicken meat is 28 mg per 100 g. Throughout the storage period, TVN levels increased in all treatments, reflecting ongoing microbial activity and protein degradation. However, samples treated with clove extract exhibited significantly lower TVN values compared to the control group, indicating the inhibitory effect of the extract on spoilage bacteria and nitrogenous compound formation. This observation aligns with findings by Fan et al. (2018), who reported a similar reduction in TVN in meat treated with oregano extract. At the end of the 7‐day storage period, the control samples reached the highest TVN values, which were significantly greater than those in treated groups (*p* < 0.01), confirming the protective role of clove extract in retarding spoilage and preserving meat quality during refrigeration.

It is noted that the control group samples exceeded the internationally accepted threshold for TVN (30 mg/100 g) on Day 7. This discrepancy between national and international standards may be attributed to several factors: first, regulatory limits set by different organizations can vary in terms of safety margins and acceptable spoilage levels. Second, variations in storage conditions, sampling methods, and TVN assay protocols can influence the results. Additionally, intrinsic variability and sensitivity of the TVN measurement method may partly contribute to observed differences.

The increase of TVN above the international threshold in the control group indicates the onset and progression of microbial spoilage and protein degradation under untreated conditions, which aligns with findings from similar studies. Importantly, treatments containing clove extract effectively inhibited the rise in TVN, maintaining levels significantly below both national and international limits during storage. This highlights the preservative and antimicrobial efficacy of clove extract in enhancing the quality and shelf life of poultry meat. Therefore, the results not only confirm the effectiveness of clove extract but also reflect differences in TVN standard limits among organizations.

### 3.7 Antimicrobial Activity in Chicken Fillet

The results of microbiological analyses are presented in Tables 8 and 9. Initial microbial loads of chicken fillet were measured as total plate count (TPC) and psychrotrophic bacteria, with values of 4.7237 ± 0.016 and 4.3868 ± 0.032 log CFU/g, respectively Abdollahzadeh et al. (2014). During the storage period, the bacterial populations in the control samples increased significantly, reaching 9.4187 ± 0.026 log CFU/g for TPC and 9.1017 ± 1.838 log CFU/g for psychrotrophic bacteria by Day 7. These values were significantly higher than those observed in treated groups (*p* < 0.01).

**TABLE 8:** The measurement Total Plate Count (log cfu/g) of chicken fillets during a 7‐day storage period.

Treatment	Storage time (day)		
	0	3	7
1	4.7237 ± 0.016 (b)	7.2570 ± 0.033 (e)	9.4187 ± 0.026 (h)
2	4.7237 ± 0.016 (b)	6.6944 ± 0.018 (d)	8.3321 ± 0.022 (g)
3	4.7237 ± 0.016 (b)	6.4732 ± 0.082 (c)	7.9565 ± 0.016 (f)
4	4.7237 ± 0.016 (b)	4.1127 ± 0.047 (a)	7.2995 ± 0.026 (e)

*Note:* Values are mean ± SD from triplicate determinations; Different letters show the statistical significant differences (*p* < 0.01).

**TABLE 9:** The measurement of Psychrotrophic bacteria (log cfu/g) of chicken fillets during a 7‐day storage period.

Treatment	Storage time (day)		
	0	3	7
1	4.3868 ± 0.032 (b)	7.0402 ± 1.060 (e)	1.838 ± 9.1017 (h)
2	4.3868 ± 0.032 (b)	6.7045 ± 0.072 (d)	0.021 ± 8.4621 (g)
3	4.3868 ± 0.032 (b)	6.4169 ± 0.105 (c)	0.046 ± 7.9019 (f)
4	4.3868 ± 0.032 (b)	4.0065 ± 0.003 (a)	0.033 ± 7.0767 (e)

*Note:* Values are mean ± SD from triplicate determinations; Different letters show the statistical significant differences (*p* < 0.01).

The treatment containing 3% clove extract demonstrated the most pronounced antimicrobial effect, effectively suppressing the growth of both TPC and psychrotrophic bacteria throughout the storage period (*p* < 0.01). This finding corroborates previous studies, such as Iheagwara (2013), who reported similar antimicrobial efficacy of clove extract. Additionally, Safari et al. (2018) observed that natural extracts could delay microbial spoilage by approximately 5 days in bighead carp (*Aristichthys nobilis*) fillet, while Ozyurt et al. (2012) reported that various plant extracts maintained lower bacterial counts during refrigerated storage.

Moreover, the 3% clove extract treatment consistently maintained the lowest bacterial counts, indicating a strong inhibitory effect on microbial proliferation compared to control samples. These results align with findings by Abdollahzadeh, Rezaei, and Hosseini (2021) and He et al. (2016), which demonstrated that antimicrobial agents significantly reduce bacterial growth in food products. Overall, samples treated with clove extract at different concentrations exhibited significantly reduced populations of both psychrotrophic bacteria and TPC compared to controls, underscoring the extract's potential as a natural preservative in extending the shelf life of chicken fillet during refrigeration.

### 3.8 Sensory Evaluation

The results indicated no significant differences in color between the control and the treated samples across Tables 10, 11, 12, 13, 14, 15. Similarly, taste and odor scores did not differ significantly between the control and other chicken fillet treatments, suggesting that the addition of clove extract did not adversely affect these sensory attributes. Regarding overall acceptability, no statistically significant differences were observed, indicating that consumer acceptance remained consistent across all treatments. However, a significant difference was found in texture (*p* < 0.05), with the chicken fillet treated with 3% clove extract receiving the highest texture scores. This suggests that the clove extract positively influenced the textural properties of the fillet, possibly by interacting with the muscle proteins or affecting moisture retention. Overall, the 3% clove extract treatment achieved the highest sensory evaluation scores, highlighting its potential to enhance certain quality attributes without compromising overall sensory acceptance.

**TABLE 10:** Friedman‐texture factor.

*N*	5
Chi‐square	9.088
df	3
Asymp. Sig.	0.028

**TABLE 11:** Texture factor ranking.

	Mean rank
Text 1	1.60
Text 2	2.30
Text 3	2.50
Text 4	3.60

**TABLE 12:** Friedman‐ color factor.

*N*	5
Chi‐square	6.308
df	3
Asymp. Sig.	0.098

**TABLE 13:** Friedman‐ aroma and odor factor.

*N*	5
Chi‐square	5.250
df	3
Asymp. Sig.	0.154

**TABLE 14:** Friedman‐ taste factor.

*N*	5
Chi‐square	4.714
df	3
Asymp. Sig.	0.194

**TABLE 15:** Friedman‐ overall acceptance Factor.

*N*	5
Chi‐square	2.833
Df	3
Asymp. Sig.	0.418

Sensory evaluation was conducted by five trained panelists who scored the samples on a scale from 1 to 5. Regarding texture, there was a statistically significant difference among the samples at the 5% significance level. Increasing the concentration of clove extract in the chicken fillets corresponded to an improvement in texture quality. The sample containing 3% extract received the highest texture score of 3.60, followed by the 2% and 1% extract samples with scores of 2.50 and 2.30, respectively. The control sample had the lowest texture score at 1.60.

In terms of color, although the chicken fillets became slightly darker as the concentration of extract increased, these differences were not statistically significant across treatments.

Regarding aroma and odor, the addition of clove extract to the samples did not cause any significant changes.

Similarly, no significant differences were observed in taste scores between the control sample and those treated with varying concentrations of clove extract.

In terms of overall acceptability, no significant differences were observed between the control sample and the treatments containing 1%, 2%, and 3% clove extract. The addition of clove extract had no statistically significant effect on sensory attributes such as color, aroma, odor, taste, or general acceptance. However, increasing the concentration of the extract showed a slight positive influence across all four sensory indicators, though not to a statistically significant extent except for texture, where the effect was significant. Higher concentrations of clove extract improved the texture of chicken fillets, with the 3% treatment receiving the highest panelist scores.

The findings of this study demonstrate that clove extract not only preserved the sensory quality of chicken fillets but also enhanced certain aspects, particularly texture. Importantly, the addition of clove extract did not result in any undesirable sensory characteristics. This contrasts with the study by Bankova and Popova (2017), where the use of 1% oregano essential oil, although effective in extending shelf life, negatively impacted sensory attributes due to its strong flavor. In the current study, clove extract was well‐accepted by panelists, likely because its flavor is familiar and commonly used in culinary applications. This compatibility with consumer taste preferences highlights the potential of clove extract as a natural preservative that supports both shelf life extension and sensory quality in meat products.

In a study conducted by Ghadiri Amrei et al. (2023) on the effect of clove essential oil in hamburgers, sensory evaluation results indicated that increasing the concentration of essential oil led to a decrease in overall acceptance, which contrasts with the findings of the present study. Conversely, Kazemeini and Aras Khalaji (2023) investigated the effect of orange peel extract on the quality of carp fillets during refrigerated storage, and their sensory evaluation showed that fillets containing 5% extract exhibited significantly better sensory attributes compared to samples with lower extract concentrations. Therefore, it can be concluded that increasing the concentration of orange peel extract enhances the sensory properties of carp fillets, a finding that supports the results of the current research.

## 4 Conclusions

The results of this study demonstrated that clove extract effectively inhibits lipid oxidation and microbial growth, while also improving certain sensory attributes, particularly texture, and extending the shelf life of chicken fillets. Among the treatments tested, the sample containing 3% clove extract showed the greatest efficacy in reducing lipid oxidation and microbial proliferation. Therefore, ethanol‐based clove extract can be considered a valuable natural preservative for maintaining the quality and prolonging the durability of chicken fillets. Furthermore, our findings suggest that combining clove extract with edible coating methods and evaluating their performance could be a promising approach to enhance antioxidant and antimicrobial effects in chicken fillets and other meat products.

## Author Contributions

**Zahra Khoshdouni Farahani:** conceptualization (equal), data curation (equal), formal analysis (equal), funding acquisition (equal), investigation (equal), methodology (equal), resources (equal), software (equal), writing – original draft (equal). **Peyman Mahasti Shotorbani:** supervision (equal), validation (equal), visualization (equal). **Afshin Akhondzadeh Basti:** project administration (equal), visualization (equal), writing – review and editing (equal). **Hassan Hamedi:** resources (equal), writing – review and editing (equal). **Bahram Hassani:** resources (equal). **Abdorreza Mohammadi Nafchi:** supervision (equal).

## Consent

Written informed consent was obtained from all study participants.

## Conflicts of Interest

The authors declare no conflicts of interest.

## Data Availability

The data may be available from the corresponding author upon reasonable request.
